# Selective inhibition by antiflamrnin-2 of thromboxane B_2_ release from isolated and perfused guinea-pig lung

**DOI:** 10.1155/S0962935192000383

**Published:** 1992

**Authors:** Lidia Sautebin, Giuseppe Cirino, Massimo Di Rosa

**Affiliations:** Department of Experimental Pharmacology University of Naples Federico II via Domenico Montesano 49 Naples 80131 Italy

## Abstract

Antiflammin-2 (AF2) is a nonapeptide corresponding to the amino acid residues 246–254 of lipocortin-1 showing anti-inflammatory activity both in vitro and in vivo. The effect of AF2 on the thromboxane B_2_ (TXB_2_) and histamine release from isolated and perfused guinea-pig lungs has been studied. AF-2 (10–100 nM) inhibited leukotriene C_4_- (LTC_4_) (3 ng) and antigen-induced (ovalbumin, 1 mg) TXB_2_ release in normal and sensitized lungs, respectively. In contrast AF-2 (100 nM) did not modify TXB_2_ release induced by histamine (5 μg) or bradykinin (5 μg) in normal lungs. Antigen-induced histamine release was not affected by 100 nM AF-2 infusion. When tested in chopped lung fragments AF-2 (0.1–25 μM) did not modify the release of histamine and TXB_2_ induced by antigen (ovalbumin, 10 μg ml^−1^) or calcium ionophore A 23187 (1 μM). Our results show that the inhibitory effect of AF-2 on TXB_2_ release is selective and depends on the stimulus applied. In this respect AF-2 mimics, at least in part, the actions of both glucocorticoids and lipocortin-1.


Research Paper

Mediators of Inflammation, 1, 251–254 (1992)

### Selective inhibition by antiflammin-2 of thromboxane $B_2$ release from isolated and perfused guinea-pig lung

Lidia Sautebin,[CA] Giuseppe Cirino and Massimo Di Rosa

Department of Experimental Pharmacology, University of Naples Federico II, via Domenico Montesano 49, 80131 Naples, Italy

[CA] Corresponding Author

**ANTIFLAMMIN-2 (AF2) is a nonapeptide corresponding to the amino acid residues 246–254 of lipocortin-1 showing anti-inflammatory activity both *in vitro* and *in vivo*. The effect of AF2 on the thromboxane $B_2$ ($TXB_2$) and histamine release from isolated and perfused guinea-pig lungs has been studied. AF-2 (10–100 nM) inhibited leukotriene $C_4$- ($LTC_4$) (3 ng) and antigen-induced (ovalbumin, 1 mg) $TXB_2$ release in normal and sensitized lungs, respectively. In contrast AF-2 (100 nM) did not modify $TXB_2$ release induced by histamine (5 μg) or bradykinin (5 μg) in normal lungs. Antigen-induced histamine release was not affected by 100 nM AF-2 infusion. When tested in chopped lung fragments AF-2 (0.1–25 μM) did not modify the release of histamine and $TXB_2$ induced by antigen (ovalbumin, 10 μg $ml^{-1}$) or calcium ionophore A 23187 (1 μM). Our results show that the inhibitory effect of AF-2 on $TXB_2$ release is selective and depends on the stimulus applied. In this respect AF-2 mimics, at least in part, the actions of both glucocorticoids and lipocortin-1.**

**Key words:** Antiflammins, Guinea-pig, Lung, Thromboxane $B_2$

### Introduction

Uteroglobin[1] and lipocortin-1[2] belong to a family of structurally related proteins referred to as annexins or lipocortins which are calcium-binding proteins.[3–5] Lipocortins are glucocorticoid-inducible proteins which, by inhibiting phospholipase $A_2$ ($PLA_2$), are believed to be involved in the suppression of prostaglandin and leukotriene synthesis associated with some aspects of the anti-inflammatory activity of corticosteroids.[6]

Recently nonapeptide fragments of uteroglobin and lipocortin-1, named antiflammins,[7] have been shown to inhibit $PLA_2$ *in vitro* and to possess anti-inflammatory activity *in vivo*.[7–9] One of these nonapeptides, antiflammin-2 (AF-2, HDMNKVLDL) corresponds to the amino acid residues 246–254 of lipocortin-1.[7] However, conflicting results have been reported concerning the biological activity of AF-2. The peptide has been shown to inhibit carrageenan-induced rat paw oedema,[9] porcine pancreatic $PLA_2$[7] and human polymorphonuclear leucocyte $PLA_2$ activity[8] as well as $PLA_2$ activity in extracts of human psoriatic epidermis.[10] In contrast, other authors have reported that AF-2 is unable to inhibit porcine $PLA_2$, eicosanoid-release by different cell types, and carrageenan-induced rat paw oedema.[11–13]

In the light of these conflicting results we decided to evaluate the biological activity of AF-2 in the isolated perfused guinea-pig lung model where human recombinant lipocortin-1 has been shown to be very active in inhibiting $LTC_4$-induced $TXB_2$ release.[14] We have also tested the effect of the peptide on histamine release in the same experimental model.

### Materials and Methods

*Animals:* Male guinea-pigs (Dunkin Hartley, 300–400 g) were used. In some experiments animals were sensitized by subcutaneous and intraperitoneal injections of equal doses (100 mg) of ovalbumin[15] and the lungs were then removed 3 weeks later.

*Mediator release from perfused lungs:* Lungs from normal or sensitized guinea-pigs were cannulated through the pulmonary artery, excised and suspended in a chamber where they were immediately perfused with oxygenated Krebs bicarbonate solution at 37°C using a Watson and Marlow 503 S peristaltic pump. The rate of perfusion was constant at 5 ml $min^{-1}$. AF-2 was infused at 0.1 ml $min^{-1}$ 30 min before stimulating $TXB_2$ or histamine release and throughout the experiment. Fractions of 5 ml, corresponding to a 1 min collection time, were collected and stored at −80°C for assay. Stimuli such as $LTC_4$ (3 ng), ovalbumin (1 mg), histamine (5 μg) or bradykinin (5 μg) were applied as a bolus injection (0.1 ml).

*Mediator release from chopped lungs:* Lungs from normal or sensitized male guinea-pig were cannulated and perfused with Krebs bicarbonate as described above. Lungs were then removed, cut into small

© 1992 Rapid Communications of Oxford Ltd pieces with a sharp blade and washed extensively. Triplicate samples (0.6 g wet weight) were incubated in Krebs bicarbonate solution at 37°C for 10 min in the presence of AF-2 (0.1–25 $\mu$M) and subsequently challenged with ovalbumin (10 $\mu$g/ml$^{-1}$ sensitized lungs) or calcium ionophore A23187 (1 $\mu$M, normal lungs). The incubation was stopped 30 min later by transferring the samples to an ice bath. Incubation media were then centrifuged (50 × *g*) and aliquots for $TXB_2$ and histamine determination immediately frozen and kept at −80°C until analysis. Lung fragments were resuspended in Krebs bicarbonate solution, boiled for 10 min and filtered. The filtrate was centrifuged (50 × *g*) and aliquots for histamine assay were kept at −80°C until analysis.

In some experiments lungs were cannulated *in situ* through the pulmonary artery and perfused with AF-2 for 30 min. The lungs were then rapidly removed, cut into small pieces and incubated and processed as described above.

*Radioimmunoassay of thromboxane $B_2$:* Thromboxane $B_2$ was measured by radioimmunoassay without prior extraction or purification as previously described[16] and expressed as ng min$^{-1}$ in perfusion experiments.

*Fluorimetric analysis of histamine:* Histamine release was measured fluorimetrically[17] and corrected for spontaneous release occurring in the absence of the inducer and expressed as $\mu$g min$^{-1}$ in the perfusion experiments.

Since it has been reported that a reduction of the biological activity of AF-2 is caused by the oxidation of the methionine residue[8] some experiments were performed in the presence of dithiothreithol (10:1). However, we could not detect any differences in AF-2 activity.

*Materials:* The composition of Krebs bicarbonate solution (mM) was the following: NaCl 118, KCl 4.7, $KH_2PO_4$ 1.2, $MgSO_4.7H_2O$ 1.17, $CaCl_2.6H_2O$ 0.25 and glucose 8.4. Other agents used were: calcium ionophore A23187 (Calbiochem), ovalbumin grade II, histamine hydrochloride, bradykinin, $TXB_2$, $LTC_4$ (Sigma); phtaldialdeyd (Fluka), radiolabelled $TXB_2$ (Amersham). AF-2 was a generous gift from Dr P. Doyle (Wellcome Foundation, Beckenham, UK). Antibody anti-$TXB_2$ was kindly supplied by Dr J. Salmon (Wellcome, UK).

### Results

*Mediator release from perfused lungs:* The basal release of $TXB_2$ from isolated perfused *normal* guinea-pig lungs was 6.35 ± 1.8 ng min$^{-1}$; $n = 12$. The basal release was not affected by 10 nM AF-2 (7.2 ± 1.1 ng min$^{-1}$; $n = 4$), 30 nM AF-2 (6.5 + 1.7 ng min$^{-1}$; $n = 4$) and 100 nM AF-2 (7.35 ± 0.8 ng min$^{-1}$; $n = 4$).

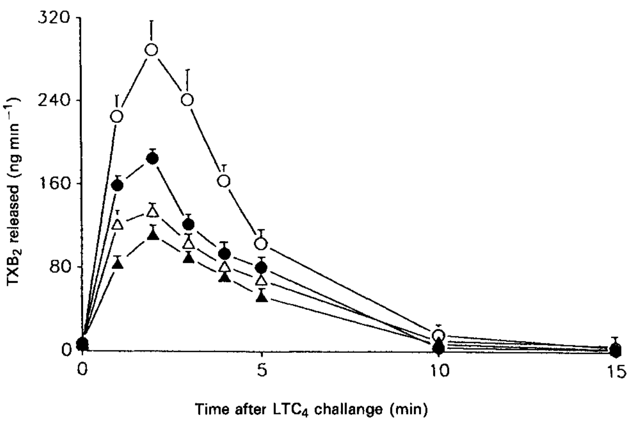

FIG. 1. Time course of $TXB_2$ release from isolated and perfused normal guinea-pig lungs challenged with a bolus injection of $LTC_4$ (3 ng). Control (○). AF-2 was infused at 10 (●), 30 (△) and 100 nM (▲). Each point represents the mean ± SE (vertical bars) of four lungs.

Normal guinea-pig lungs challenged with a bolus injection of 3 ng $LTC_4$ released large amounts of $TXB_2$ (Fig. 1). The peak response was observed 2 min after the challenge (289 ± 6 ng min$^{-1}$; $n = 4$), thereafter the $TXB_2$ release rapidly declined to a low level after 10 min (16 ± 10 ng min$^{-1}$; $n = 4$).

When the lung was infused with AF-2 the $LTC_4$-induced $TXB_2$ release was greatly reduced throughout the time course of the response (Fig. 1). The peak response was reduced by the peptide in a concentration-related fashion as inhibition of 36%, 54% and 61% were induced by 10, 30 and 100 nM infusion of AF-2.

In contrast, neither the time course nor the peak response of the $TXB_2$ release induced by histamine (5 $\mu$g) or bradykinin (5 $\mu$g) in *normal lungs* were affected by infusion of AF-2 up to 100 nM. In fact, the peak of $TXB_2$ release from lungs challenged with histamine (75 ± 2.8 ng min$^{-1}$; $n = 4$) was unaffected by 100 nM AF-2 infusion (82 ± 2 ng min$^{-1}$; $n = 4$), as the peak of $TXB_2$ release from lungs challenged with bradykinin (78 ± 13 ng min$^{-1}$; $n = 4$).

However the antigen-induced release of $TXB_2$ from sensitized lungs was reduced by 100 nM AF-2 (Fig. 2A). The peak release, which was ten-fold higher than that obtained with other stimuli (635 ± 39 ng min$^{-1}$; $n = 6$), was significantly inhibited by 32% (431 ± 8.7 ng min$^{-1}$; $n = 6$). Thromboxane $B_2$ release from sensitized unchallenged lungs (14.5 ± 1.6 ng min$^{-1}$; $n = 4$) was not affected by 100 nM infusion AF-2 (13.4 ± 1.1 ng min$^{-1}$; $n = 4$).

Antiflammin-2 infusion (100 nM) did not modify either the peak response or the time course of the antigen-induced histamine release from sensitized lungs (Fig. 2B).

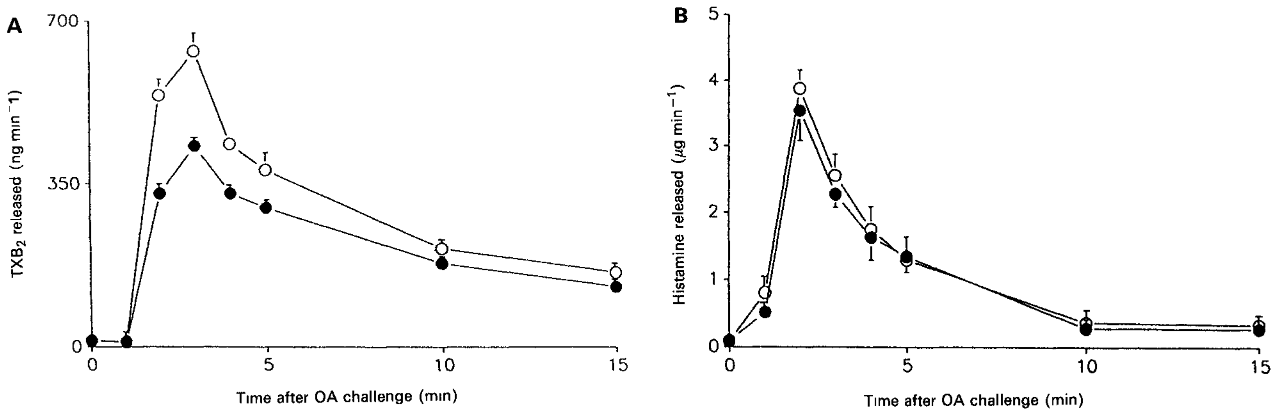

FIG. 2. Time course of $TXB_2$(A) and histamine (B) release from isolated and perfused sensitized guinea-pig lungs challenged with a bolus injection of antigen (ovalbumin 1 mg). Control (○); infusion of AF-2 100 nM (●). Each point represents the mean ± SE (vertical bars) of six lungs.

*Mediator release from chopped lungs:* In another set of experiments we evaluated the effect of AF-2 infusion on the release of $TXB_2$ and histamine from chopped guinea-pig lungs. The mediators were released from normal lungs by 1 $\mu$M calcium ionophore A23187 and by antigen (10 $\mu g\, ml^{-1}$ ovalbumin) from sensitized lungs. None of the AF-2 concentrations tested (0.1–25 $\mu$M) was able to significantly modify $TXB_2$ and histamine released induced by these agents. Similar results were obtained when lungs were perfused *in situ* with AF-2 at the above concentrations and fragments subsequently challenged with the same stimuli (data not shown).

### Discussion

These data show the ability of AF-2 to inhibit the $TXB_2$ release induced by $LTC_4$ or antigen in *normal and sensitized* guinea-pig lungs respectively. However, when other stimuli, such as histamine and bradykinin were used, no inhibition of $TXB_2$ release was observed.

Human recombinant lipocortin-1 (annexin-1) when infused through normal guinea-pig lung preparation inhibited $LTC_4$-induced $TXB_2$ release.[14] Glucocorticoids have been shown to inhibit $TXB_2$ release from guinea-pig isolated perfused lungs challenged by different stimuli such as leukotrienes, histamine or antigen while they did not prevent $TXB_2$ generation induced by bradykinin.[18]

For these reasons we studied the effect of the nonapeptide, referred to as antiflammin-2,[7] corresponding to lipocortin-1 sequence 246–254, on $TXB_2$ generation from guinea-pig lungs challenged with different stimuli in order to compare its biological activity to the effect of glucocorticoids and lipocortin-1.

Our results suggest that the inhibitory activity of AF-2 on $TXB_2$ release is dependent on the challenge used and mimics, at least in part, the action of both glucocorticoids and lipocortin-1. In fact AF-2, like glucocorticoids, significantly inhibits $TXB_2$ release induced by $LTC_4$ and it is unable to block the releasing action of bradykinin in normal lungs. In this respect it is interesting to observe that dexamethasone has an inhibitory activity on the bradykinin-induced eicosanoid release from the inflamed lungs while it is ineffective in normal lungs.[19] The reason for the lack of activity of both glucocorticoids and AF-2 in normal lungs is not clear.

It has been suggested that separated $PLA_2$ pools may exist or that a phospholipase C linked pathway could be involved.[18,20] The main difference between AF-2 and dexamethasone was observed when the lung was stimulated with histamine. In fact AF-2 was unable to block the histamine-induced $TXB_2$ release in normal lungs, which has been reported to be inhibited by glucocorticoids.[18] We found that AF-2 was unable to inhibit the antigen-induced histamine release from sensitized lungs. This observation is consistent with previous results reported by us suggesting that another glucocorticoid-induced protein (vasocortin) different from lipocortin[21] is responsible for the glucocorticoid inhibition of histamine release.[22]

The release for the conflicting results reported by different authors on the biological activity of AF-2 is not clear. In fact, the mode of action of lipocortins and lipocortin-derived peptides as $PLA_2$ inhibitors it is not yet understood. Thus until now it has not been clearly demonstrated if the $PLA_2$ inhibition depends on a direct interaction between the enzyme and the inhibitor[6] or it is due to the binding of the inhibitor to the substrate.[23] Another aspect which could affect the biological activity of the peptide might be its oxidation possibly occurring during the experimental procedures. In fact it has been suggested that AF-2 might be inactivated by spontaneous oxidation of its methionine residue in the position 3 and consequently it might be effective only in the presence of a reducing agent.[8] In this respect we did not observe any significant difference in the inhibition of $TXB_2$ release by AF-2 in the presence or absence of dithiothreitol.

Furthermore the inhibitory action of AF-2 on $TXB_2$ generation seems to be dependent not only on the stimulus but also on the experimental model used. Indeed, the peptide was completely ineffective when assayed on $TXB_2$ release from chopped lungs.

Corticosteroids are very powerful drugs in asthma therapy but their mechanism is not yet clearly understood and their clinical use mostly empirical. Antiflammin-2 could be used as a useful tool in exploring the mechanism through which glucocorticosteroids could affect the airway regulatory mechanisms.

### References

1. Levin SW, Butler JD, Schumaker UK, Wightman PD, Mukherjee AB. Uteroglobin inhibits phospholipase $A_2$ activity. *Life Sci* 1986; **38**: 1813–1819.
2. Wallner BP, Mattaliano RS, Hession C, *et al.* Cloning and expression of human lipocortin, a phospholipase $A_2$ inhibitor with potential anti-inflammatory activity. *Nature* 1986; **320**: 77–81.
3. Di Rosa M, Flower RJ, Hirata F, Parente L, Russo Marie F. Nomenclature announcement. Anti-phospholipase proteins. *Prostaglandins* 1984; **28**: 441–443.
4. Crumpton MS. Protein terminology tangle. *Nature* 1990; **345**: 212.
5. Geisow MJ, Ali SM, Boustead C. Structures and function of a supergene family of calcium and phospholipid binding proteins. In: Melli M, Parente L, eds. *Cytokines and lipocortins in inflammation and differentiation.* Chichester: Wiley-Liss, Chichester, 19; 111–121.
6. Flower RJ. Lipocortin and the mechanism of action of the glucocorticoids. *Br J Pharmacol* 1988; **94**: 987–1015.
7. Miele L, Cordella-Miele E, Facchiano A, Mukherjee AB. Novel anti-inflammatory peptides from the region of highest similarity between uteroglobin and lipocortin-1. *Nature* 1988; **335**: 726–720.
8. Camussi G, Tetta C, Bussolino F, Baglioni C. Anti-inflammatory peptides (antiflammins) inhibit synthesis of platelet-activating factor, neutrophils aggregation and chemiotaxis and intradermal inflammatory reactions. *J Exp Med* 1990; **171**: 913–927.
9. Ialenti A, Doyle PM, Hardy GN, Simpkin DSE, Di Rosa M. Anti-inflammatory effects of vasocortin and nonapeptide fragments of uteroglobin and lipocortin-1 (antiflammins). *Agents & Actions* 1990; **29**: 48–49.
10. Cartwright PH, Ilderton E, Sowden SM, Yardley HS. Inhibition of phospholipase $A_2$ in extracts of lesion free psoriatic epidermis by the nonapeptide HDMNKVLDL which corresponds to lipocortin-1 residues 246–254. *Br J Dermatol* 1990; **122**: 277–278.
11. Hope WC, Patel BJ, Bolin DR. Antiflammin-2 (HDMNKVLDL) does not inhibit phospholipase $A_2$ activities. *Agents & Actions* 1991; **34**: 77–80.
12. van Binsbergen J, Slotboom AJ, Aarsman AJ, De Haas GH. Synthetic peptide from lipocortin-1 has no phospholipase $A_2$ inhibitory activity. *FEBS* 1989; **247**: 293–297.
13. Marki F, Pfeischifter J, Rink H, Wiesenberg I. Antiflammins: two nonapeptide fragments of uteroglobin and lipocortin-1 have no phospholipase $A_2$-inhibitory and antiinflammatory activity. *FEBS* 1990; **264**: 171–174.
14. Cirino G, Flower RJ, Browning JL, Sinclair LK, Pepinsky RB. Recombinant human lipocortin-1 inhibits thromboxane release from guinea-pig isolated perfused lung. *Nature* 1987; **328**: 270–278.
15. Engineer DM, Piper PJ, Sirois P. Interaction between the release of SRS-A and prostaglandins. *Br J Pharmacol* 1976; **57**: 460–461P.
16. Salmon JA. A radioimmunoassay for 6-keto prostaglandin F1α. *Prostaglandins* 1978; **15**: 383–391.
17. Shore PA, Burkhalter A, Cohn VH. A method for the fluorimetric assay of histamine in tissues. *J Pharmacol Exp Ther* 1959; **127**: 182–186.
18. Blackwell G, Flower RJ, Nijkamp F, Vane JR. Phospholipase $A_2$ activity of guinea-pig isolated perfused lungs: stimulation and inhibition by anti-inflammatory steroids. *Br J Pharmacol* 1978; **62**: 79–89.
19. De Nucci G, Astbury P, Read N, Salmon JA, Moncada S. Release of eicosanoids from isolated lungs of guinea-pig exposed to pure oxygen: effect of dexamethasone. *Eur J Pharmacol* 1986; **126**: 11–20.
20. Robinson C, Hoult J. Evidence for functionally distinct pools of phospholipase responsible for prostaglandin release from perfused guinea-pig lung. *Eur J Pharmacol* 1980; **64**: 333–339.
21. Sautebin L, Carnuccio R, Ialenti A, Di Rosa M. Lipocortin and vasocortin: two species of anti-inflammatory proteins mimicking the effects of glucocorticoids. *Pharmacol Res* 1991; **24**: 1–12.
22. Carnuccio R, Di Rosa M, Ialenti A, Iuvone T, Sautebin L. Selective inhibition by vasocortin of histamine release by dextran and concanavalin-A from rat peritoneal cells. *Br J Pharmacol* 1989; **98**: 32–34.
23. Davidson FF, Dennis EA. Biological relevance of lipocortins and related proteins as inhibitors of phospholipase $A_2$. *Biochem Pharmacol* 1989; **38**: 3645–3641.

**Received 12 May 1992; accepted in revised form 16 June 1992**
